# The influence of context representations on cognitive control states

**DOI:** 10.1186/s41235-022-00443-0

**Published:** 2022-10-18

**Authors:** Reem Alzahabi, Erika Hussey, Nathan Ward

**Affiliations:** 1 Center for Applied Brain and Cognitive Sciences, Tufts University, Medford, USA; 2 U.S. Army Combat Capabilities Development Command Soldier Center, Natick, USA; 3 Department of Psychology, Tufts University, Medford, USA; 4 Department of Liberal Arts, Kettering University, Flint, MI 48504 USA

**Keywords:** Control state, Proactive, Reactive, Context, Task-switching

## Abstract

Cognitive control operates via two distinct mechanisms, proactive and reactive control. These control states are engaged differentially, depending on a number of within-subject factors, but also between-group variables. While research has begun to explore if shifts in control can be experimentally modulated, little is known about whether context impacts which control state is utilized. Thus, we test if contextual factors temporarily bias the use of a particular control state long enough to impact performance on a subsequent task. Our methodology involves two parts: first participants are exposed to a context manipulation designed to promote proactive or reactive processing through amount or availability of advanced preparation within a task-switching paradigm. Then, they complete an AX-CPT task, where we assess immediate transfer on preferential adoption of one control mode over another. We present results from a Pilot Study that revealed anecdotal evidence of proactive versus reactive processing for a context manipulation using long and short preparation times. We also present data from a follow-up Registered Experiment that implements a context manipulation using long or no preparation times to assess if a more extreme context leads to pronounced differences on AX-CPT performance. Together, the results suggest that contextual representations do not impact the engagement of a particular control state, but rather, there is a general preference for the engagement of proactive control.

## Introduction

The human cognitive system exhibits a remarkable ability to adjust thoughts in pursuit of goal-directed behavior. This processing, generally referred to as cognitive control, involves the active maintenance of contextual information as well as appropriate adjustments in responding such that one is able to perform tasks in face of competing sources of information (Badre & Wagner, 2007; Miller & Cohen, 2001). The dual-mechanisms of control (DMC) framework is one account that has operationalized how such control is instantiated (Braver, 2012). Under this framework, cognitive control operates in two primary modes: proactive and reactive control. Proactive control involves the active maintenance of goals, while reactive control occurs in response to changing environmental demands (Braver, 2012). While proactive control is efficient because it allows for preparation of upcoming events, it is thought to be mentally demanding and requires the sustained activation of goal-relevant information. Conversely, reactive control supports correction and resolves interference on an as-needed basis, and is therefore inherently transient in nature and thought to require minimal cognitive resources (Gonthier et al., 2016). Each control state is accessed depending on particular circumstances or environmental demands. Ultimately, exerting cognitive control involves the dynamic shifting between control states in order to achieve an optimization of goal attainment.

Despite the fact that implementing a particular cognitive control state may be optimal in a given set of circumstances, research suggests that there is considerable variability in the ability to engage each control state, both across populations as well as within-individuals (Braver, 2012). For instance, cognitive decline has been associated with a tendency to become more reliant on reactive control modes. Aging is associated with a reduced ability to engage proactive control thereby shifting the balance in favor of reactive control (Braver et al., 2001). Similarly, young children engage cognitive control reactively, but develop the ability to engage proactive control throughout early childhood (Chevalier et al., 2015). Clinical populations, including individuals with schizophrenia, as well as Alzheimer-type dementia patients, struggle to spontaneously implement proactive control (Barch et al., 2001; Braver et al., 2005). In contrast, individuals with high working memory capacity have an increased tendency toward exerting proactive control (Redick, 2014). Even so, within individuals, a number of affective and motivational factors are known to influence the engagement of different cognitive control states (Chiew & Braver, 2014; Dreisbach, 2006). For instance, state anxiety inhibits one’s ability to engage proactive control while concurrently increasing the use of reactive control (Yang, Miscovich, and Larson, 2018).

The current literature investigating the deployment of control states has flourished in recent years, and in turn, has contributed to the identification and classification of characteristics that impact which control state will be engaged, and under which circumstances. However, generally, the primary focus has been on special populations (e.g., clinical, aging populations), individual difference factors (e.g., working memory capacity, fluid intelligence), or on instances in which one deviates from their normal baseline (e.g., state anxiety). While these investigations are crucial to understanding the underpinnings of cognitive control, a more complete and nuanced understanding of cognitive control relies on the extension of this work to representative instances in typical populations. More specifically, identifying contextual (i.e., task and environmental) characteristics that influence when a particular control state is engaged will help generalize our understanding of cognitive control states.

To this end, one study investigated the role of information accessibility and found that altering participants’ amount of available goal-consistent information changes the amount of proactive or reactive control used (Burgess & Braver, 2010). Specifically, proactive control mechanisms were utilized to a greater extent when more interference was expected. Furthermore, another study found that altering the ratio of incongruent-to-congruent trials in a Stroop task leads to changes in how conflicting information is monitored, a state that the authors argue impacts the amount of proactive or reactive control used (Bugg & Crump, 2012). Related work on conflict adaptation demonstrates fluctuations in control states as a function of stimulus compatibility (Mansfield et al., 2012; Suzuki & Shinoda, 2015). These studies reported that reactive control was heavily engaged in overcoming activation of incompatible stimulus–response mappings.

Shifts in cognitive control mode have also been observed when working memory load is manipulated across conditions, such that high-load conditions are associated with the engagement of more reactive control states (Speer et al., 2003; Maki-Marttunen, Hagen, & Espeseth, 2019). In extension of this work, a number of recent studies have addressed whether cognitive control states can be experimentally modulated. One investigation reported that individuals could be systematically biased toward and away from the utilization of proactive control, through techniques such as strategy training or no-go manipulations (Gonthier et al., 2016). Overall, a number of approaches have demonstrated that proactive and reactive control states are flexibly deployed, or induced, as a function of the availability of information.

### Context manipulation

In the current investigation, we asked whether contextual factors mediate the deployment of different control states. Are individuals more likely to engage a particular control state in a specific environment or within a specific context? More specifically, do contextual factors *robustly* impact the activation of control states, such that the cognitive control state adopted in one task generalizes to a subsequent task? Generally, context processing allows individuals to internally represent patterns of environmental cues, and in turn, these cues can be utilized to exert control over behavioral responses (Rush et al., 2006). Context processing includes the formation of an internal representation of context and the maintenance of these representations over time, which includes updating the representations to mirror changes in the environment (Braver & Cohen, 2001; Braver et al., 2002). Context representations can be generated from the presentation of a prior stimulus or from the result of earlier processing and are particularly useful for guiding future behavior. Exerting control over thoughts and behavior requires internal representation, maintenance, and updating of context information (Braver & Cohen, 2000). Our aim was to identify whether contextual factors impact an individual’s likelihood to engage proactive or reactive control.

In this study, we manipulated context using a task-switching paradigm. There are a couple of ways context can be manipulated in a task-switching paradigm, given that, in this paradigm the cue-stimulus interval (CSI) reflects the amount of time one has to prepare for a task. The context can vary such that the length of the CSI varies, so there is either a “short” or “long” time to prepare for a task. Alternatively, the more extreme instance is that the CSI can be present or not, so that there is either no time to prepare for a task, or that preparation time is made available. The prediction is that preparation time via CSI length will place participants into relatively proactive (as in the case of longer CSIs) or reactive (for shorter CSIs) control states. Indeed, Chevalier and colleagues (2015) present a similar manipulation in a sample of young children; we aimed to adopt this paradigm and extend this work to an adult sample.

### Proactive/reactive control transfer

A body of research on information-transfer demonstrates that exposure to one task can influence performance on a subsequent task that relies on similar neurocognitive resources or procedures (Dahlin et al., 2008). In fact, a number of studies have demonstrated local transfer of an executive function task within the same experimental session (e.g. Persson et al., 2007, 2013; Surrey et al., 2017; Weidler, Dey, & Bugg, 2018). For instance, practicing a task with high-interference demands activates cognitive control resources for a period of time such that performance on other interference tasks is impacted systematically (Persson et al., 2007, 2013). As such, here we addressed if the context representation emerging from completing a task varying in preparation time allotted has impacts on whether one is more likely to subsequently engage a particular control state on another task.

To measure the sustained use of proactive and reactive cognitive control, participants completed the AX-CPT task immediately after the task-switching task. In a CPT task, participants are required to make speeded responses to items they see in a continuous sequence. In the AX-CPT version of this task, participants identify a target for an X that immediately follows an A (Barch et al., 1997). The A cue prompts subjects to prepare for an X target, which speeds response times for target identification. To delineate the strategic use of proactive and reactive control in this task, participants encounter non-AX pairs, such as AY, BX, and BY trials. Because 80% of trials are either AX targets and BY non-targets, participants become accustomed to preparing a target response to items immediately following an A and preparing a non-target response to items immediately following a B. In the case of AY trials, the presence of the A cue activates proactive control in anticipation of the X target, but because the subsequent item is a Y, response times slow down and this is used to index the strength of the initiated proactive control. In the case of BX trials, a B cue indicates that the subsequent item is not a target, yet X is highly associated with a target response, because of AX trial experience. As such, response times slow down and this is used to index reactive control. Lastly, the BY trials are used as a control condition because neither item is associated with a target response, and thus, no slowdown is assumed. In sum, the AX-CPT paradigm allows us to measure the degree to which individuals preferentially use one cognitive control state over the other after being exposed to a context manipulation in a task-switching paradigm (Braver, 2012). We predicted that context representations that allow more time to prepare (through longer CSIs) would lead to a higher likelihood of activating proactive states and that this would have concomitant effects on proactive control usage in the AX-CPT relative to contexts where less preparation time is allowed, which would follow a more reactive control profile in terms of AX-CPT performance.

In the sections that follow, we first present the details of a General Methodology to test for context representation effects on cognitive control states. Then, we present the design and results of a pilot study, which inform design decisions for a second experiment. We end by presenting the Registered Experiment, along with the data and interpretation of the results.

## General methodology

The experimental design includes a session containing a Task-Switching Paradigm with varying preparation times intended to induce proactive or reactive states, followed immediately by the AX-CPT Paradigm, where proactive and reactive performance can be assessed.

### Task-switching paradigm

Participants completed versions of a task-switching paradigm programmed using E-prime software (Schneider et al., 2002). In every version of the paradigm, participants switched between two tasks across trials: a number (odd/even) and letter (consonant/vowel) classification task. A descriptive cue (“Classify Number”/“Classify Letter”) was used to denote the to-be-classified stimulus. The cue was randomly selected on each trial, yielding approximately 50% switch trials, in which the type of stimulus to be classified changes from one trial to the next (e.g., Classify Number then Classify Letter), and 50% repeat trials, in which the type of stimulus to be classified remains the same from one trial to the next (e.g., Classify Number then Classify Number). The stimulus consists of a randomly selected number (2, 4, 6, 8, 3, 5, 7, or 9)/letter (g, k, m, r, a, e, i, or u) pair presented in the center of the screen (e.g., “2G”). Participants were instructed to respond using “1” and “2” on the number keypad (1 = “even”/“consonant”; 2 = “odd”/“vowel”). Response labels were provided on the bottom of the monitor screen on every trial. After making a response, participants saw a blank screen for 132 ms. Participants completed a total of 96 trials.

Each version reflected one context manipulation, which was manipulated in terms of amount of preparation allowed (e.g., long, short, or no time). In the instructions, participants were informed of the amount of time they have to prepare for each stimulus in advance. Below, we describe the differences between versions presented during the Pilot Study and those for the Registered Experiment.

### AX-CPT paradigm

We used a version of the AX-CPT adapted from Gonthier et al. (2016) and programmed using E-prime software. Each trial began with a cue, which was a letter (any letter except X, K, or Y) appearing in the center of the screen for 1000 ms. A blank inter-stimulus interval of 4000 ms followed. After the inter-stimulus interval, the probe appeared, which was a letter (any letter except A, K, or Y) appearing in the center of the screen for 500 ms. After the probe, a row of asterisks appeared in the center of the screen during a 1000 ms inter-trial interval. Participants were instructed to press the target button with the middle finger of their right hand as quickly as possible whenever they observed an A cue followed by an X probe, and to press the non-target key with the index finger of the right hand as quickly as possible whenever they observed any other letter pair. Responses to the probe stimuli were recorded within a time frame of 1500 ms.

The proportions of trial types were as follows: 40% of the trials in each task block consisted of an A followed by an X (AX trials), 10% of the trials in each block consisted of an A followed by a letter other than X (AY trials), 10% of the trials in each block consisted of a letter other than A followed by an X (BX trials), and 40% of the trials in each block consisted of a letter other than A followed by a letter other than X (BY trials) (Gonthier et al., 2016; Richmond et al., 2015). Participants completed four blocks of 50 trials, resulting in a total of 200 trials (80 AX, 20 AY, 20 BX, 80 BY). Trials within each block were presented randomly. Prior to completing the experimental trials, participants viewed a demonstration of multiple types of trials with the correct response presented. They also completed 10 practice trials before the experimental trials. Identical AX-CPT paradigms were used in the Pilot Study and Registered Experiment.

Error rates and average response times (RTs) for correct responses were recorded for each of the four trial types (AX, AY, BX, and BY) in the AX-CPT paradigm. In addition, three indices reflecting the relative use of proactive control were computed: d’-context, A-cue bias, and the Proactive Behavioral Index (PBI) (see Gonthier et al., 2016; Stanislaw & Todorov, 1999). Based on signal detection theory, d’-context and A-cue bias reflect participants’ ability to use contextual information from the cue to select the probe response and the tendency to make a target response following the A cue, regardless of the identity of the probe. The PBI quantifies the preference to deploy proactive control by combining correct RT and error rate performance on AY and BX trials: (AY − BX)/(AY + BX).

### Design and procedure

**Fig. 1:**
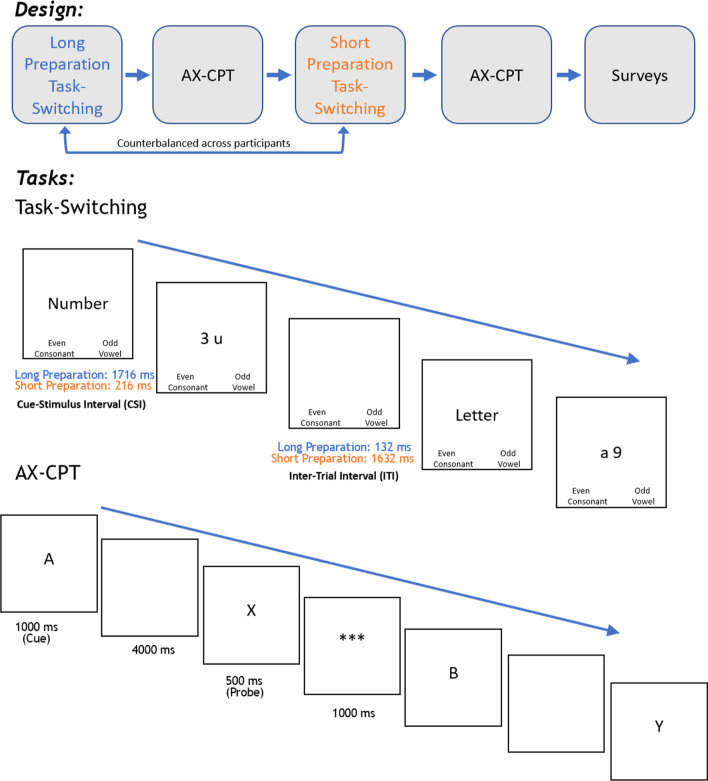
Task design for the Pilot Study. Cue timings for the long preparation condition are indicated in blue text and in orange text for the short preparation condition. Inter-trial interval denotes time period after participant makes a response and before the cue for the next trial is presented (i.e., response-cue interval)

**Fig. 2:**
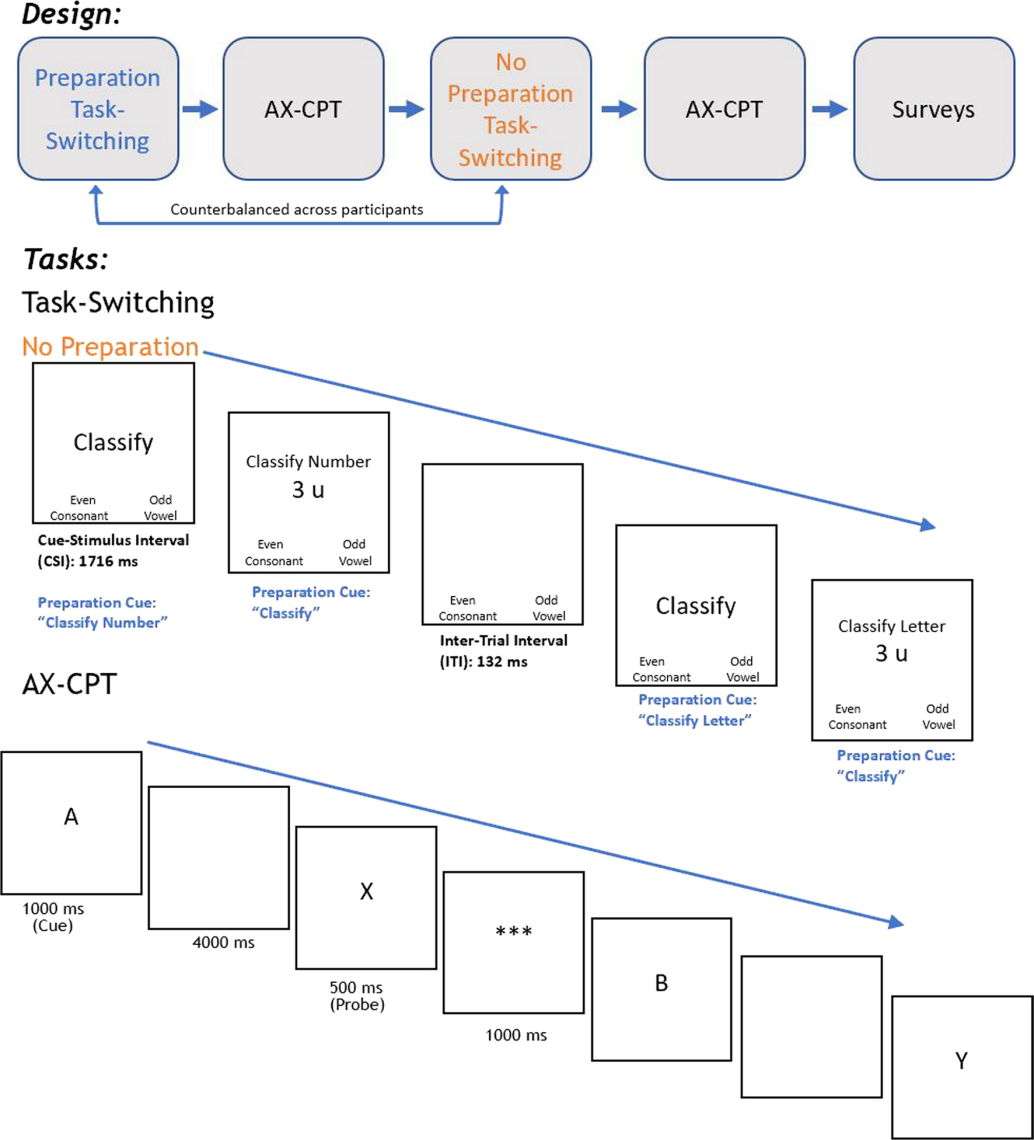
Task design for Registered Experiment. Cues for the preparation condition are indicated in blue text. Inter-trial interval denotes time period after participant makes a response and before the cue for the next trial is presented (i.e., response-cue interval)

Eligibility criteria to participate in the study included being over the age of 18; normal or correct-to-normal vision; and no self-reported history of concussion, Traumatic Brain Injury, and/or neuropsychological conditions. After participants provided informed consent, they sat approximately 50 cm from a Dell monitor to complete two versions of the task-switching paradigm varying in preparation allowed, each followed by the AX-CPT paradigm (see Figs. 1, 2). The order of the task-switching versions was counterbalanced across participants. After completing each version of the task-switching paradigm, as well as the AX-CPT task twice, participants completed surveys, including the Media Use Questionnaire (Baumgartner et al., 2016) and the Brief Sensation Seeking Scale (BSSS; Stephenson et al., 2003), which have been associated with task-switching performance, and they were offered a debriefing form.

## Pilot study

We conducted a Pilot Study with 77 university undergraduates (52 females; mean age = 19.4 years) who participated for course credit. Participants completed a block-by-block design as described in the General Methodology above.

### Context manipulation

As Fig. 1 depicts, two versions of the Task-Switching paradigm were presented in order to manipulate context representation: In the extended preparation version, participants were presented with a descriptive cue for 1716 ms (long CSI), while the cue only appeared for 216 ms (short CSI) in the shorter preparation version. After making a response, participants saw a blank screen for 132 ms in the long CSI version and 1632 ms in the short CSI version. Because we prioritized having a consistent trial time across versions (1848 ms), the inter-trial-intervals were different across versions.

### Pilot study results

**Fig. 3:**
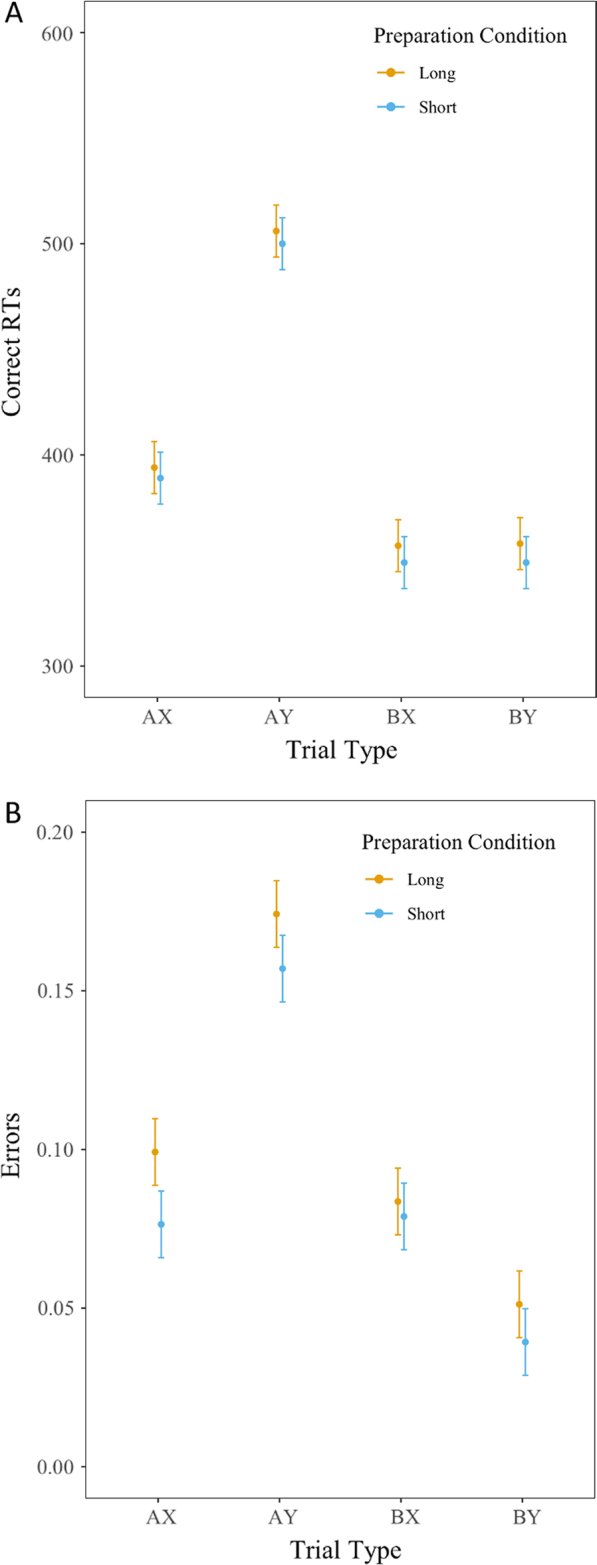
Average median correct response times (ms) (**A**) and error rates (**B**) in the AX-CPT as a function of AX-CPT trial type and preparation condition (long or short) in the Pilot Study. Error bars = standard error of the mean (SEM)

To assess the effectiveness of our task-switching manipulation, we conducted a repeated-measures ANOVA on the task-switching RT data, which revealed main effects of CSI, *F*(1, 74) = 10.11, *p* < 0.01 (long preparation < short preparation) and trial type, *F* (1, 74) = 16.08, *p* < 0.01 (repeat < switch). The error data also revealed a marginally significant main effect of CSI, *F*(1,74) = 3.57, *p* = 0.06 (long preparation < short preparation), significant main effect of trial type *F*(1,74) = 25.45, *p* < 0.01 (repeat < switch), and significant CSI × trial type interaction, *F*(1,74) = 11.86, *p* < 0.01. This analysis suggests that participants performed differently on the task-switching blocks depending on the preparation time they were provided. After this manipulation check, any participants with overall error rates greater than 40% on any of the Task-Switching trial types (switch, repeat) or AX-CPT trial types (AX, AY, BX, BY) were excluded from the analysis. Using the data that survive this criterion (*n* = 64), we conducted all AX-CPT analyses using a generalized linear model that included Preparation Condition (long vs. short) and Trial Type (AX, AY, BX, BY) as factors. For correct AX-CPT RTs, the main effect of preparation condition was non-significant, *F*(1,63) = 2.01, *p* = 0.16, partial *η*^2^ = 0.03, indicating similar response times on AX-CPT items following both preparation time manipulations. As expected, the main effect of trial type was significant, *F*(3,189) = 181.67, *p* < 0.01, partial *η*^2^ = 0.74. BY and BX trials led to faster RTs compared to AX trials (*p* < 0.05), which all led to faster RTs compared to AY trials (*p* < 0.01). Furthermore, there was no condition by trial type interaction, *F*(3,189) = 0.12, *p* = 0.95, partial *η*^2^ < 0.01 (See Fig. 3, Panel A; Table 1).

**Table 1:** Descriptive statistics for the AX-CPT as a function of preparation condition (long or short) in the Pilot Study

Trial type	Long preparation	Short preparation
*Median correct response times* (ms)		
AX	394 (76.1)	389 (67.7)
AY	506 (97.3)	500 (83.1)
BX	357 (134)	349 (114)
BY	358 (106)	349 (91.6)
*Error rates*		
AX	0.10 (0.09)	0.08 (0.07)
AY	0.17 (0.10)	0.16 (0.11)
BX	0.08 (0.09)	0.08 (0.08)
BY	0.05 (0.06)	0.04 (0.06)
*Combined measures*		
PBI RTs	0.19 (0.10)	0.19 (0.09)
PBI error rates	0.33 (0.37)	0.30 (0.41)
D’ context	2.85 (0.93)	2.94 (0.81)
A-cue bias	0.24 (0.28)	0.24 (0.23)

Shown are average values with standard deviations in parentheses

For AX-CPT error rates, the main effect of preparation condition (long or short) was also non-significant, *F*(1,63) = 2.19, *p* = 0.14, partial *η*^*2*^ = 0.03, indicating similar error rates on AX-CPT items following both preparation time manipulations. As expected, the main effect of trial type (AX, AY, BX, BY), was significant, *F*(3,189) = 71.73, *p* < 0.01, partial *η*^2^ = 0.53. BY trials led to lower error rates than AX and BX trials (*p* < 0.05), which all had lower error rates compared to AY trials (*p* < 0.01). Furthermore, there was no condition by trial type interaction, *F*(3,189) = 0.67, *p* = 0.57, partial *η*^2^ = 0.01 (See Fig. 3, Panel B; Table 1).

The three indices reflecting the use of proactive control (*d*’-context, A-cue bias, and the Proactive Behavioral Index) were also computed and revealed no significant differences as a function of context (long vs. short), *p*s > 0.4. PBI RT, PBI error rate, *d*’-context, and A-cue bias scores were all significantly greater than zero following both the long and short preparation contexts (*p*s < 0.001).

### Pilot study conclusion

Generally, participants engaged proactive control, as indicated by PBI RT and PBI error scores that were significantly greater than zero following both the long and short preparation contexts. However, task-switching preparation time (long vs. short) did not impact the engagement of a particular control state. Following both short and long preparation contexts, participants equally engaged proactive and reactive control, as indexed by AX-CPT performance. In sum, this pilot data suggests that transient contextual representations in one task do not impact which cognitive control state is utilized in another task. However, in addition to conducting standard null hypothesis testing, we also conducted statistical tests using Bayes Factors, to allow us to quantify the strength of evidence of the null hypothesis compared to the alternative. Bayes factor indicated moderate evidence in favor of the null hypothesis for RTs (BF_10_ = 0.15) and error rates (BF_10_ = 0.60), suggesting that an element of preparation time may not be impacting the engagement of particular cognitive states. We reasoned that it may be possible that providing *any* period of preparation time, regardless of whether it is “long” or “short,” is inducing a state of proactive control. As indicated earlier, proactive control on the AX-CPT is indicated by the preparation of a target response for the “A” cue and a non-target response for a non- “A” cue. Thus, presenting a preparatory cue in the context of a cued task-switching paradigm may be facilitating preparation on the AX-CPT, which is perhaps associated with a more proactive control profile across both preparation contexts.

### Pilot study discussion

As such, it is crucial to test the more extreme context manipulation that differentiates between whether a preparatory period exists or not. Indeed, it has been suggested that the emergence of proactive control is contingent on the preparatory period between the task cue and the impending stimulus (Bugg & Braver, 2016; Chevalier et al., 2015). This preparatory period can be changed in length (as we tested in our pilot study), but it can also be changed in its availability. Data from this investigation will determine if contextual representations generally do not impact the engagement of a particular control state, or if contextual representations impact the engagement of control states, but only if they fall within a particular set of parameters.

## Registered experiment

To this end, the goal of the Registered Experiment was to assess the extent to which contextual representations (preparation time) impact the deployment of a particular control state. Here, we asked if presenting the cue simultaneously with the to-be-classified stimulus impacts the likelihood that proactive control is engaged. Alternatively, does a context in which there is no advanced preparation time cause the deployment of a more reactive control state?

We conducted the registered experiment with 48 university undergraduates (26 females; mean age = 21.3 years) who participated for course credit. Participants completed a block-by-block design as described in the General Methodology, and under the following context manipulation.

### Context manipulation

The experimental design manipulated the presence of a preparation interval in the Task-Switching paradigm (preparation vs. no preparation; see Fig. 2). In the no preparation version, a neutral cue (“Classify”) was presented for 1716 ms, followed by a descriptive cue (e.g., “Classify Letter”) along with the to-be-classified stimulus. In the instructions, participants were informed that they have no time to prepare for the task in advance and that they simply must wait for the descriptive cue to appear with the stimulus so as to know which task to perform. In the preparation version, the descriptive cue (e.g., “Classify Letter”) was presented for 1716 ms, followed by a neutral cue (e.g., “Classify”) along with the to-be-classified stimulus. In this condition, participants were told that they have an opportunity to prepare for the upcoming task, as they will be provided with a descriptive cue prior to the appearance of the stimulus. In fact, they will not be provided with the cue alongside the stimulus, and as such, task preparation is required to successfully complete the task accurately and quickly. In addition to presenting an extreme context manipulation, this design equated for timing differences in the preparation time manipulation found in the Pilot Study. In the Pilot Study, our goal was to only manipulate the time given to prepare, but given difference timings across the two CSIs, there may have been differences in boredom, or fatigue, across the two conditions. Also, to reduce any elements of task complexity due to stimulus–response mappings, participants were instructed to respond using keyboard responses labeled with “even”/“consonant” and “odd”/ “vowel.”

### Data analysis and predictions

Again, to assess the effectiveness of our task-switching manipulation, we conducted a repeated-measures ANOVA on the task-switching RT data (Preparation condition x Trial type), which revealed main effects of CSI, *F*(1, 47) = 61.4, *p* < 0.01 (preparation < no preparation) and trial type, *F* (1, 47) = 34.8, *p* < 0.01 (repeat < switch), and a significant CSI x trial type interaction, *F*(1,47) = 15.9, *p* < 0.01. The error data also revealed significant main effects of CSI, *F*(1,47) = 8.88, *p* < 0.01 (preparation > no preparation), and trial type *F*(1,47) = 9.87, *p* < 0.01 (repeat < switch). This analysis suggests that participants performed differently on the task-switching blocks depending on whether or not they were provided with preparation time. It also indicates that more errors were being committed in the preparation condition compared to the no preparation condition.

Any participants with overall error rates greater than 40% on any of the Task-Switching trial types (switch, repeat) or AX-CPT trial types (AX, AY, BX, BY) were excluded from the analysis (*n* = 10). Using the data that survive this criterion, we conducted analyses on the error rates and median correct RTs using a generalized linear model to assess the effects Preparation Condition (Preparation vs. No Preparation) and Trial Type (AX, AY, BX, BY). We also conducted analyses on PBI Error Rate, PBI RTs, D’-Context, and A-Cue Bias measures. Because these are measures that combine AX-CPT trial types, the generalized linear model included only Preparation Condition as a factor.

We expected to find a significant interaction of Preparation Condition and Trial Type for Error Rate and Correct RTs, and expect a series of selective effects on AX-CPT performance. Specifically, following the preparation contexts, we should observe that participants are better at preparing a target response for the “A” cue and a non-target response for a non- “A” cue in the AX-CPT compared to cases following no-preparation contexts. In contrast, following the no-preparation contexts, we should observe that participants are selectively retrieving information when the probe appears in the AX-CPT. This would translate to better BX (shorter RTs and lower error rates), and worse AY performance (longer RTs and higher error rates) following the preparation contexts compared to cases following the no-preparation contexts.

We also expected to find a significant main effect of Preparation Condition for PBI Error and PBI RT scores, D’-Context, and A-Cue Bias. A more proactive control state should manifest as higher values on all four measures.

### Sample size estimate

We conducted a power analysis using G*Power 3.1 (Faul et al., 2007) to estimate the sample size needed to detect effects on the AX-CPT. Previous work testing the influence of strategies on AX-CPT performance (Edwards et al., 2010; Gonthier et al., 2016) report effect sizes of the Strategy Condition by Trial Type interactions ranging from partial eta squared = 0.14 to 0.39 with an average effect size of 0.27. A required sample size of 48 was determined for an effect of this magnitude with alpha = 0.05 and power = 0.95.

### Registered experiment results

**Fig. 4:**
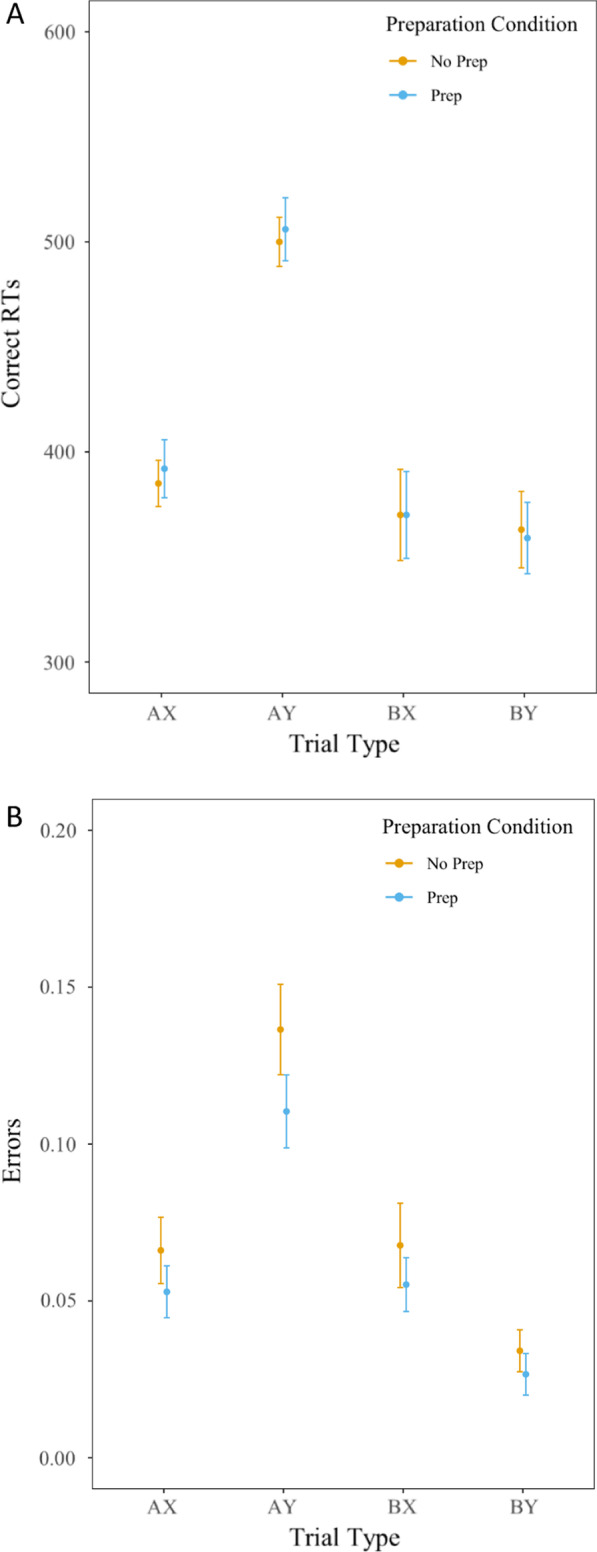
Average median correct response times (ms) (**A**) and error rates (**B**) in the AX-CPT as a function of AX-CPT trial type and preparation condition (Prep or No Prep) in the Registered Experiment. Error bars = standard error of the mean (SEM)

We conducted all AX-CPT analyses using a generalized linear model that included Preparation Condition (Prep vs. No Prep) and Trial Type (AX, AY, BX, BY) as factors. For AX-CPT RTs, the main effect of preparation condition was non-significant, *F*(1,47) = 0.08, *p* = 0.79, partial *η*^2^ < 0.01, indicating similar response times on AX-CPT items following both preparation time manipulations. As expected, the main effect of trial type was significant, *F*(3,141) = 111.34, *p* < 0.01, partial *η*^2^ = 0.70. BY and BX trials led to faster RTs compared to AX trials (*p* < 0.05), which all led to faster RTs compared to AY trials (*p* < 0.01). Furthermore, there was no condition by trial type interaction, *F*(3,141) = 0.44, *p* = 0.73, partial *η*^2^ < 0.01 (See Fig. 4, Panel A; Table 2).

**Table 2:** Descriptive statistics for the AX-CPT as a function of preparation condition (Prep or No Prep)

Trial type	Preparation	No preparation
*Median correct response times* (ms)		
AX	392 (95.5)	385 (76.1)
AY	506 (104)	500 (80.8)
BX	370 (143)	370 (151)
BY	359 (118)	363 (126)
*Error rates*		
AX	0.05 (0.06)	0.07 (0.07)
AY	0.11 (0.08)	0.14 (0.10)
BX	0.06 (0.06)	0.07 (0.09)
BY	0.03 (0.05)	0.03 (0.05)
*Combined measures*		
PBI RTs	0.17 (0.10)	0.17 (0.10)
PBI error rates	0.25 (0.38)	0.35 (0.35)
D’ context	3.26 (0.75)	3.18 (0.92)
A-cue bias	0.25 (0.27)	0.28 (0.27)

Shown are average values with standard deviations in parentheses

For AX-CPT error rates, the main effect of preparation condition (no prep or prep) was also non-significant, *F*(1,47) = 2.89, *p* = 0.10, partial *η*^2^ = 0.06, indicating similar error rates on AX-CPT items following both preparation time manipulations. As expected, the main effect of trial type (AX, AY, BX, BY), was significant, *F*(3,141) = 48.54, *p* < 0.01, partial *η*^2^ = 0.51. BY trials led to lower error rates than AX and BX trials (*p* < 0.05), which all had lower error rates compared to AY trials (*p* < 0.01). Furthermore, there was no condition by trial type interaction, *F*(3,141) = 0.67, *p* = 0.57, partial *η*^2^ = 0.01 (See Fig. 4, Panel B; Table 2).

The three indices reflecting the use of proactive control (*d*’-context, A-cue bias, and the Proactive Behavioral Index for errors and RTs) were also computed and revealed no significant differences as a function of preparation condition (prep vs. no prep), *p*s > 0.15. PBI RT, PBI error rate, *d*’-context, and A-cue bias scores were all significantly greater than zero following both the no preparation and preparation contexts (*p*s < 0.01).

We also conducted an additional between-subjects analysis, to assess for any potential order effects (Prep first vs. No Prep first) that could result from counterbalancing. Repeated-measures ANOVAs revealed no main effects of order for RTs, *F*(1,46) = 1.88, *p* = 0.18, or for errors *F*(1,46) = 0.07, *p* = 0.80, and no interaction between Trial Type and order for both RTs, *F*(3,138) = 0.74, *p* = 0.53, and errors, *F*(3,138) = 0.18, *p* = 0.91. One-way ANOVAs for *d*’-context, A-cue bias, and the Proactive Behavioral Index for errors and RTs, also revealed that order was not significant for any of these variables (all *p*s > 0.14).

### Registered experiment conclusion

Generally, task-switching preparation time (prep vs. no prep) did not impact the engagement of a particular control state. Participants did engage proactive control, as indicated by PBI RT and PBI error scores that were significantly greater than zero following both the long and short preparation contexts. However, we expected to find a significant interaction of Preparation Condition and Trial Type for Error Rate and Correct RTs, in addition to a series of selective effects on AX-CPT performance. Following both no preparation and preparation contexts, participants equally engaged proactive and reactive control, as indexed by AX-CPT performance. In sum, this additional data also suggests that transient contextual representations in one task do not impact which cognitive control state is utilized in another task.

## General discussion

Across two context manipulations, our results suggest that contextual representations do not impact the engagement of a particular control state. We tested the impact of manipulating the length of the preparatory period, in addition to a more extreme context manipulation that differentiated between whether a preparatory period existed or not. We found that providing *any* period of preparation time, regardless of whether it is “long” or “short,” and providing no preparation period, were all associated with the engagement of proactive control. Overall, this suggests a general bias to engage proactive control mechanisms, rather than a selective bias for one control state.

In line with our Pilot Study analysis, we again conducted statistical tests using Bayes Factors, to allow us to quantify the strength of evidence of the null hypothesis compared to the alternative. Bayes factor indicated moderate evidence in favor of the null hypothesis for RTs (BF_10_ = 0.11) and no evidence in favor of the alternative hypothesis for error rates (BF_10_ = 1.04), suggesting that preparation time is likely not impacting the engagement of a particular cognitive state.

These findings are consistent with work that suggests a developmental shift in the engagement of proactive control (Chevalier et al., 2015). Specifically, older children show a robust ability to engage proactive control, more so than younger children who only engage proactive control when reactive control is made more difficult. Furthermore, older adults engage a reactive control pattern, unlike younger adults who demonstrate a bias for proactive control (Braver et al., 2009). Generally, adults flexibly engage the most adaptive control mode depending on various factors, such as working memory capacity.

More generally, it is also likely that the context representation emerging from completing a task-switching task is subsequently encouraging the selective engagement of proactive control. In the context of task-switching, research has demonstrated that task cues could facilitate proactive control in both young and older adults (Chang et al., 2020). Also, trial-by-trial shifts between proactive and reactive control have been demonstrated in task-switching, such that left superior parietal activity covaries with the magnitude of switching costs (Braver et al., 2003). In fact, task-switching requires the maintenance of two (or more) task-sets, and both repeat and switch trials within mixed-task blocks can be cognitively demanding, specifically for young children. Change in task-switching performance across the lifespan, particularly as proactive control becomes less demanding throughout childhood and into adolescence, is more so driven by performance increases on both switch and repeat trials within mixed-task blocks, rather than performance increases that are selectively different for switch and repeat trials (Chevalier et al., 2015). This suggests that task-switching in and of itself may be encouraging the engagement of proactive control more so than reactive control.

Future work investigating the relationship between contextual representations and cognitive control states could expand on the current work as well as address potential limitations of the current investigation. First, conducting a similar study with a larger sample size could help provide more solid evidence in favor of either the null or alternative hypothesis. Furthermore, given that our task-switching manipulation resulted in more errors in the preparation condition compared to the no preparation condition, a larger sample size could assess how this may be impacting the effects on the control state being engaged. In addition, alternate versions of the AX-CPT, such as those with no-go trials implemented (e.g., Gonthier et al., 2016), could be more sensitive for detecting the effect of context manipulations, particularly because they lead to less of a baseline bias towards proactive control. Finally, it would be beneficial to gather data on a variety of individual difference factors, such as working memory capacity or general control state bias, so as to identify how these factors might interact with context manipulations. In fact, context manipulations may be more or less effective in some sub-groups of participants, particularly considering that high working memory capacity individuals may be associated with a natural tendency to engage proactive control (Rosales et al., 2021).

## Data Availability

All data and analysis pipelines from our study, as well as a copy of the stage 1 registered report, are stored on a repository hosted by the Center for Open Science at https://osf.io/78h35/ to enable transparency, repeatability, and future use of data.

## Abbreviations

CSI: Cue-stimulus interval
RT: Reaction time
AX-CPT: AX continuous performance task
PBI: Proactive behavioral index
